# Deciphering the Action of Neuraminidase in Glioblastoma Models

**DOI:** 10.3390/ijms241411645

**Published:** 2023-07-19

**Authors:** Nathalie Baeza-Kallee, Raphaël Bergès, Victoria Hein, Stéphanie Cabaret, Jeremy Garcia, Abigaëlle Gros, Emeline Tabouret, Aurélie Tchoghandjian, Carole Colin, Dominique Figarella-Branger

**Affiliations:** 1 Aix Marseille Univ, CNRS, INP, Inst Neurophysiopathol, 13005 Marseille, France; nathalie.baeza@univ-amu.fr (N.B.-K.); raphael.berges@univ-amu.fr (R.B.); victoria.hein96@gmail.com (V.H.); abigaelle.gros@ap-hm.fr (A.G.); emeline.tabouret@gmail.com (E.T.); aurelie.tchoghandjian@univ-amu.fr (A.T.); carole.colin@univ-amu.fr (C.C.); 2 ChemoSens Platform, Centre des Sciences du Goût et de l’Alimentation, InstitutAgro, CNRS, INRAE, Université de Bourgogne Franche-Comté, 21000 Dijon, France; stephanie.cabaret@inrae.fr; 3 APHM, CHU Timone, Service d’Anatomie Pathologique et de Neuropathologie, 13005 Marseille, France; jeremy.garcia@ap-hm.fr; 4 APHM, CHU Timone, Service de Neurooncologie, 13005 Marseille, France

**Keywords:** glioblastoma, cancer stem cells, A2B5, ganglioside, targeted treatment, neuraminidase

## Abstract

Glioblastoma (GBM) contains cancer stem cells (CSC) that are resistant to treatment. GBM CSC expresses glycolipids recognized by the A2B5 antibody. A2B5, induced by the enzyme ST8 alpha-N-acetyl-neuraminide alpha-2,8-sialyl transferase 3 (ST8Sia3), plays a crucial role in the proliferation, migration, clonogenicity and tumorigenesis of GBM CSC. Our aim was to characterize the resulting effects of neuraminidase that removes A2B5 in order to target GBM CSC. To this end, we set up a GBM organotypic slice model; quantified A2B5 expression by flow cytometry in U87-MG, U87-ST8Sia3 and GBM CSC lines, treated or not by neuraminidase; performed RNAseq and DNA methylation profiling; and analyzed the ganglioside expression by liquid chromatography–mass spectrometry in these cell lines, treated or not with neuraminidase. Results demonstrated that neuraminidase decreased A2B5 expression, tumor size and regrowth after surgical removal in the organotypic slice model but did not induce a distinct transcriptomic or epigenetic signature in GBM CSC lines. RNAseq analysis revealed that *OLIG2*, *CHI3L1*, *TIMP3*, *TNFAIP2*, and *TNFAIP6* transcripts were significantly overexpressed in U87-ST8Sia3 compared to U87-MG. RT-qPCR confirmed these results and demonstrated that neuraminidase decreased gene expression in GBM CSC lines. Moreover, neuraminidase drastically reduced ganglioside expression in GBM CSC lines. Neuraminidase, by its pleiotropic action, is an attractive local treatment against GBM.

## 1. Introduction

Glioblastoma (GBM), *IDH* wildtype, is the most common malignant primary tumor of the central nervous system [1]. Its prognosis remains dismal after the standard of care, which relies on surgery followed by radiotherapy and chemotherapy [2]. The median overall survival is around 15 months with no significant improvement in spite of a plethora of clinical trials [3]. There is therefore an urgent need for new therapeutic approaches targeting relevant targets. Three main factors are responsible for the resistance to treatment: the blood–brain barrier, the tumor-propagating microenvironment, and the occurrence of tumor-initiating cells or cancer stem cells (CSC), which are resistant to cell death and drive relapse.

Several cell surface markers have been reported in GBM CSC, including, among others, CD133 and A2B5 [4,5,6,7,8,9]. The A2B5 monoclonal antibody, originally prepared against chicken embryo retina cells [10], reacts with gangliosides that contain a trisialo sequence (i.e., GT3 and alkali-labile O-acetyl GT3, GQ1c and GP1c) [11,12,13]. Gangliosides are glycosphingolipids composed of a ceramide lipid tail attached through glycosidic linkage to a glycan headgroup containing one or more sialic acid residues. According to the Svennerholm classification, gangliosides can be classified in four series (0-, a-, b-, and c-series) based on the number of sialic acid residues (from 0 to 3) linked to the inner galactose residue [14], the A2B5 antibody recognizing gangliosides of the c-series (for review [15]).

Ganglioside biosynthesis involves sequential activities of distinct glycosyltransferases and sialyltransferases. The ST8Sia family is known to catalyze the production of α2,8-linked sialic acid chains according to the acceptor specificity of glycoproteins and gangliosides [16]. ST8Sia3 has been proposed to deliver α2,8-triSia and polySia units onto glycoproteins in vitro [17], and we have shown that ST8Sia3 strongly increased A2B5 expression at the cell surface of U87-MG and U251-MG cell lines [18].

In human GBM, A2B5 and CD133 expression distinguishes three cell populations: A2B5−/CD133−, A2B5+/CD133+, and A2B5+/CD133−. Although the A2B5− cell population is not able to generate tumors when they are injected into the brains of nude rats or mice, the two other populations generate tumors [7,8]. By using MACS to isolate and characterize A2B5+ and A2B5− fractions from fresh human GBM, we showed that A2B5+ but not A2B5− cells have cancer stem cell properties: they can form spheroids, are self-renewing, and are able to differentiate towards the neural, astrocytes and oligodendrocytes when placed in differentiating media [8]. Furthermore, we and others have generated GBM CSC lines by isolating A2B5+ cells from fresh human samples [8,9,19,20]. Importantly, the number of A2B5+ cells is maintained through serial passages and is recorded in almost all cells after the third passage [9]. In order to decipher the functional role of the A2B5 epitope in human GBM, we used various cell models that express high or low A2B5 levels. To this end, the ST8Sia3 gene was stably overexpressed by lentiviral infection or silenced by using shRNA technology in two GBM cell lines expressing mild (U251-MG, 50.25% ± 3.06) and low (U87-MG, 17.5% ± 0.96) levels of A2B5 immunoreactivity. ST8Sia3 mRNA was significantly increased in ST8Sia3-overexpressing cells in comparison to the wild-type cell lines and the shST8sia3 cell lines. When grafted orthotopically in mice, extended survival was observed with sh-ST8sia3 cell lines in comparison with the ST8Sia3 cell lines. Since the lentiviral delivery of shST8Sia3 in two CSC lines (GBM6 and GBM9) expressing a high level of A2B5 stopped their growth, we used neuraminidase administration to remove the A2B5 epitope at the cell surface of CSC lines and observed impaired cell survival, proliferation and self-renewal [18].

The aim of the present study was to better understand the effect of neuraminidase administration at the cellular level to tackle human GBM CSC. To achieve this goal, first, we quantified A2B5 expression at the cell surface of our cell models before and after neuraminidase administration; second, we used an organotypic three-dimensional model to further characterize the action of neuraminidase; third, we searched for a transcriptomic and epigenetic signature induced by neuraminidase administration as well as changes in ganglioside expression.

## 2. Results

### 2.1. A2B5 Expression in Native Cell Lines and after Neuraminidase Administration

**Figure 1:**
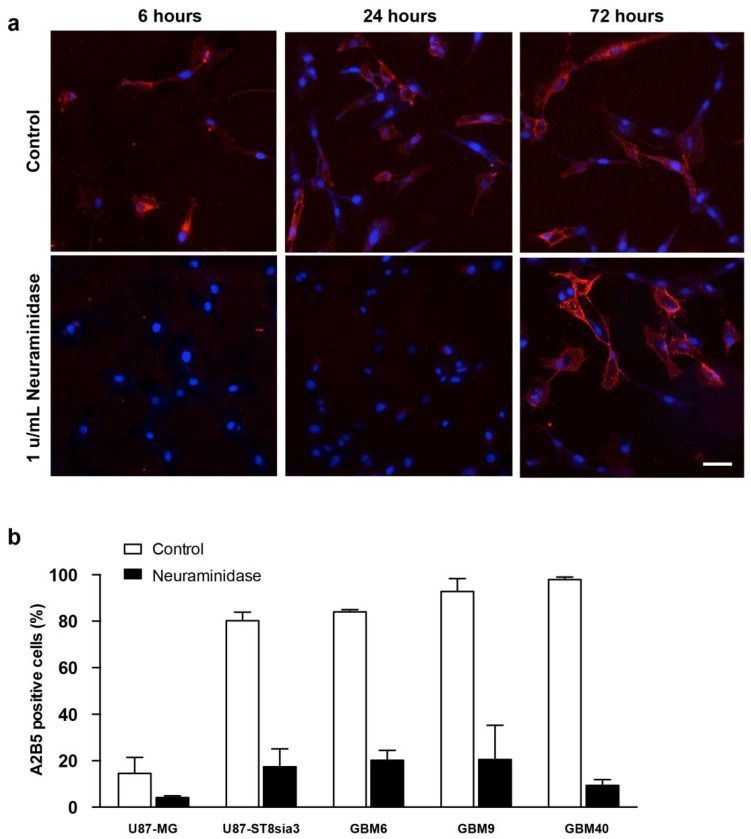
Immunofluorescence detection of A2B5 (red) expression on U87-ST8Sia3 cells before and after neuraminidase administration at 6, 24 and 72 h. Hoechst staining of the cell nuclei (blue) is also shown. Scale bar = 10 μm (**a**). Quantification of A2B5 expressing by flow cytometry in GBM cell lines treated with neuraminidase or vehicle (control) for 24 h (**b**).

Using immunofluorescence, we evaluated the expression of A2B5 on U87-ST8Sia3 cells before and 6, 24 or 72 h after neuraminidase administration at a concentration of 1 u/mL (EC50) (Figure 1a). According to our previous results [18], strong A2B5 immunoreactivity was recorded on most of the U87-ST8Sia3 cells. Interestingly, the expression was abolished as early as 6 h after neuraminidase administration and reappeared 72 h later.

Then, using flow cytometry, we quantified the percentage of cells expressing A2B5 with and without neuraminidase administration (at EC50) in U87-MG, U87-ST8Sia3 and GBM CSC lines (Figure 1b). In non-treated cells, we recorded 14.6% ± 6.9 of cells expressing A2B5 in the U87-MG cell line and 80.2% ± 3.6 in the U87-ST8Sia3 cell line resulting from the stable infection of U87-MG cells with the ST8Sia3 gene. In contrast to the U87-MG cell line, the percentage of A2B5-expressing cells in CSC lines (GBM9, GBM6, GBM40) accounts for 84.0% ± 0.2 (GBM6), 92.8% ± 5.6 (GBM9) and 97.9% ± 1.1 (GBM40). The percentage of A2B5-expressing cells was drastically reduced, but not abolished, 24 h after neuraminidase administration in all cell lines accounting for 4.2% ± 0.1 (U87-MG), 17.3% ± 7.8 (U87-ST8Sia3), 20.2% ± 4.2 (GBM6), 20.5% ± 14.7 (GBM9) and 9.4% ± 2.5 (GBM40) (Figure 1b).

### 2.2. Neuraminidase Administration Strongly Decreased A2B5 Expression Tumor Size and Regrowth after Surgical Removal in a GBM Organotypic Slice Model

In order to monitor the action of neuraminidase in an integrated ex vivo model, we set up a GBM organotypic slice model. Briefly (for details, see Methods), we prepared spheroïds of U87-ST8Sia3-GFP, GBM6-GFP and GBM9-GFP lines. These spheroïds were then grafted on 250 μm-thick brain sections prepared from adult athymic female nude mice. The organotypic co-cultures were maintained at 37 °C, and at days 1, 3, 6, 8, 10 and 15 post-spheroid implantation, treated with neuraminidase at EC50 or PBS as control.

**Figure 2:**
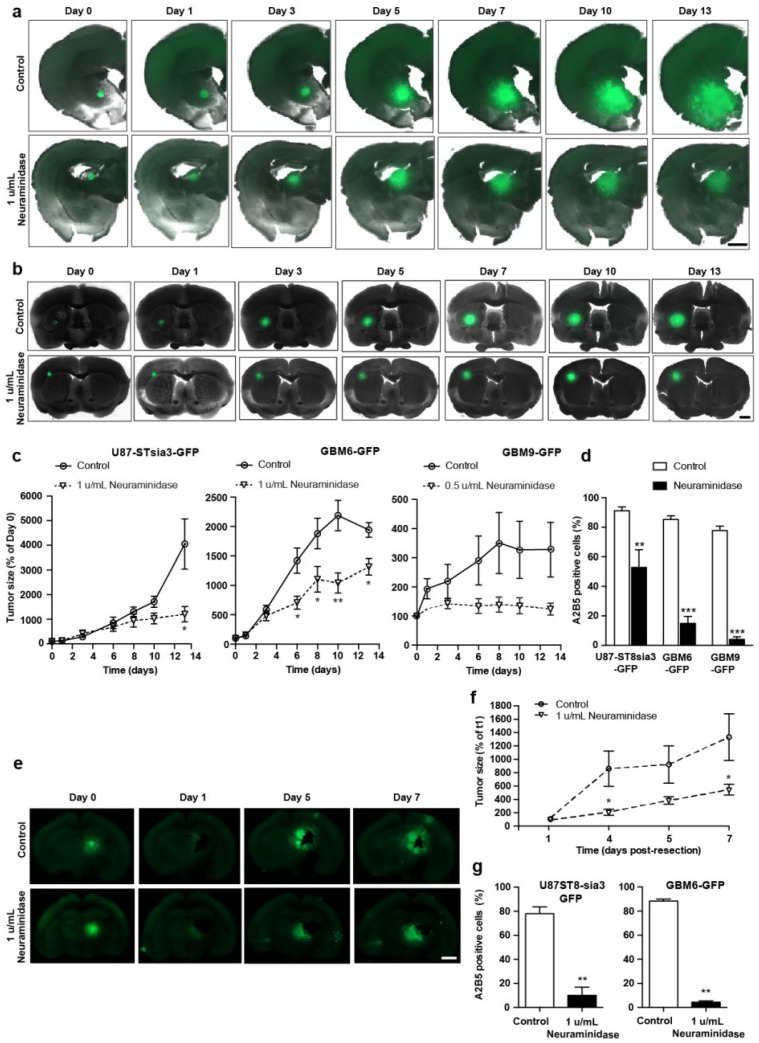
Representative fluorescent images of coronal brain slices co-cultured with U87-ST8Sia3-GFP (**a**) and GBM6-GFP spheroids (**b**) treated with neuraminidase at EC50 or vehicle (control) at days 1, 3, 6, 8 and 10 post-spheroid implantation (day 0). Scale bar = 1000 µm. Quantification of tumor size obtained by the implantation of U87-ST8Sia3-GFP, GBM6-GFP and GBM9-GFP spheroids in organotypic slices treated with neuraminidase at EC50 or vehicle (control) (**c**). Flow cytometry analysis of A2B5 of U87-ST8Sia3-GFP, GBM6-GFP and GBM9-GFP cells, isolated from tumor resection at day 16 post-spheroid implantation (**d**). Representative fluorescent images of tumor regrowth after tumor resection (**e**). Coronal brain slices cocultured with U87-ST8Sia3-GFP spheroids were treated with neuraminidase at EC50 or vehicle (control) at days 1, 4 and 6 after tumor resection (day 0). Bar = 1000 µm. Quantification of size of the tumor regrowth (U87-ST8Sia3-GFP and GBM6-GFP) in the organotypic slices treated with neuraminidase at EC50 or vehicle (control) (**f**). Flow cytometry analysis of A2B5 of U87-ST8Sia3-GFP and GBM6-GFP cells, isolated from tumor at day 7 post tumor resection, after treatment with neuraminidase at EC50 or vehicle (control) (**g**). At least three independent experiments were performed. *** *p* < 0.001, ** *p* < 0.01, * *p* < 0.05.

Importantly, we observed a strong decrease in the size of tumors generated by the spheroids when the slices were treated with neuraminidase, compared to control (−70.2% ± 6.5, −32.2% ± 7.3 and −62.3% ± 6.2 at day 13 post-spheroid implantation in U87-ST8Sia3-GFP, GBM6-GFP and GBM9-GFP, respectively) (Figure 2a–c). However, the tumor area remained stable from 5–7 days to 13 days of the experiment, suggesting that although neuraminidase strongly impaired the growth of the tumor, it does not induce a regression.

We also confirmed that neuraminidase administration strongly decreased A2B5 at the cell surface of the tumors formed after the implantation of spheroids generated by the U87-ST8Sia3-GFP, GBM6-GFP and GBM9-GFP cell lines (Figure 2d). The number of A2B5-expressing cells was 91.2% ± 2.6 in tumors generated by U87-ST8Sia3-GFP spheroids in vehicle-treated slices (controls), compared to 52.9% ± 11.9 after neuraminidase administration. Similar results were observed in tumors generated after GBM6-GFP and GBM9-GFP spheroids with a number of A2B5-expressing cells of 85.3% ± 2.4 in GBM6-GFP and 77.8% ± 2.9 in GBM9-GFP control slices, although it was drastically reduced after neuraminidase (14.9% ± 4.7 and 4.1% ± 1.8, respectively).

In addition, we also used this organotypic slice model to mimic tumor resection and explore whether the administration of neuraminidase affected tumor regrowth. When neuraminidase was added after the surgical removal of the tumors generated by U87-ST8sia3-GFP or GBM6-GFP spheroids, we observed the impairment of tumor regrowth compared to control (−59.1% ± 6.2 at day 7 post-tumor resection) (Figure 2e,f). This decrease in tumor size was associated with a reduction in A2B5 expression compared to control, with a number of A2B5-expressing cells of 78.0% ± 5.6 in U87-ST8Sia3-GFP and 88.5% ± 1.3 in GBM6-GFP tumors from control slices and 9.9% ± 6.8 and 3.1% ± 0.7 after neuraminidase administration (Figure 2g).

### 2.3. Neuraminidase Administration Did Not Induce a Distinct Transcriptomic Signature in CSC Lines

**Figure 3:**
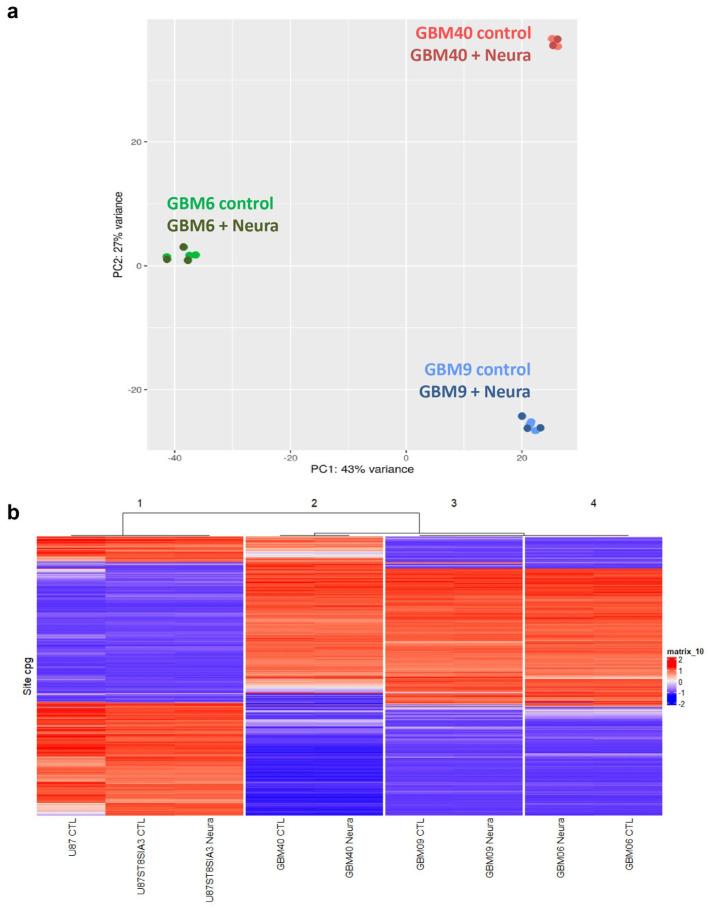
Absence of modification of transcriptomic signature and epigenetic signature after neuraminidase administration (Neura) to CSC (GBM6, GBM9, GBM40) and U87-ST8Sia3 cell lines. Principal component analysis (PCA) on native and treated CSC lines (GBM6, GBM9, GBM40): samples group together by cell lineage, not by treatment conditions (**a**). Unsupervised hierarchical clustering of DNA-methylation profiling data of 9 samples: native control (CTL) cell lines (GBM6, GBM9, GBM40, U87-MG, U87-ST8Sia3) and their treated counterparts group together by cell lineage, not by treatment conditions (**b**).

In order to search for a transcriptomic signature induced by neuraminidase administration, we performed RNA-seq from GBM6, GBM9 and GBM40 CSC lines before and after 24 h of neuraminidase administration. We observed that, when performing principal component analysis (PCA) on all the native and treated CSC lines, samples were grouped together by cell lineage (Figure 3a), and when performing PCA on each CSC line separately (native and treated samples), samples were grouped slightly together by batch. Moreover, each CSC line shared with its neuraminidase-treated counterpart almost the same transcriptomic signature; therefore, this approach did not allow the identification of a distinct transcriptomic signature induced by neuraminidase administration.

### 2.4. DNA Methylation-Profiling Demonstrated Strong Differences in Epigenetic Signature in U87 Cell Lines Versus CSC Lines but no Distinct Signature Was Induced by Neuraminidase

For each cell line, the DNA methylation class (MC) and the calibrated score (CS) according to the v11.b4 and v12.5 versions of the DKFZ classifier were provided (Supplementary Table S1). With the v11.b4 version of the classifier, GBM6, GBM9 and GBM40 displayed an MC of GBM, *IDH*-wildtype with a CS of 0.80, 0.86 and 0.76, respectively. With the v12.5 version of the classifier, GBM6 and GBM9 received an MC of GBM, *IDH*-wildtype subclass RTKI with a CS of 0.47 and 0.52, respectively, whereas GBM40 displayed an MC of GBM, *IDH*-wildtype subclass RTKII with a CS of 0.44 CS. In contrast, no MC was provided by the DKFZ classifier for U87-MG and U87-ST8Sia3 cell lines.

Furthermore, no change in DNA methylation profiling was induced in each cell line after neuraminidase administration. Unsupervised hierarchical clustering demonstrated two major groups: the first one was made by the U87-MG cell line and its derivatives and the second by the CSC lines. We observed that each cell line clustered with its neuraminidase-treated counterpart. Moreover, it was obvious that the DNA methylation profile of the U87-MG cell line and its derivatives was strikingly different from that of the CSC lines (Figure 3b).

In addition, the copy number variation (CNV) profile of the CSC lines differs from that of the U87-MG cell line. All CSC lines exhibited a chromosome 7 gain and *CDKN2A* homozygous deletion, and GBM 40 also showed chromosome 6 and 10 deletions. *MYC* amplification was also recorded in all CSC lines (Supplementary Figure S1a–d).

### 2.5. ST8Sia3 Overexpression in U87-MG Cell Line Induced a Specific Transcriptomic Signature

**Figure 4:**
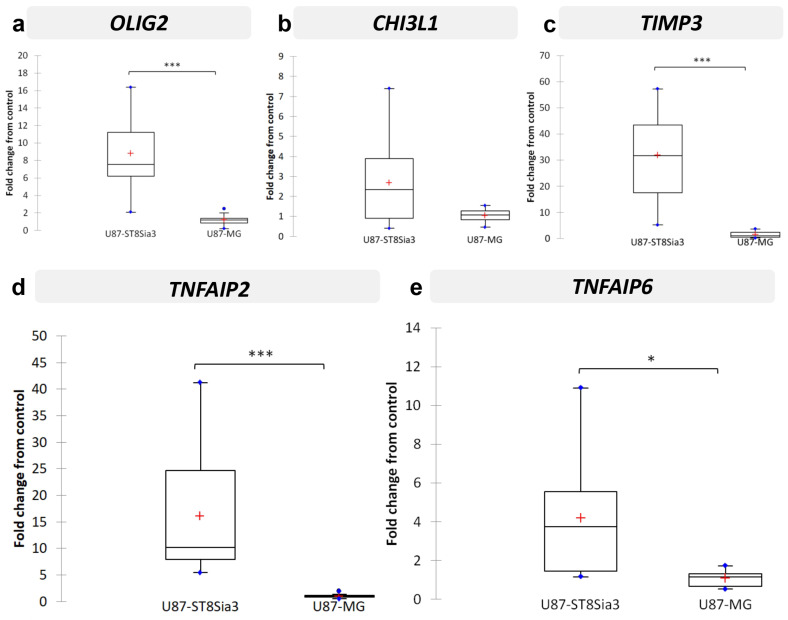
Validation by RT-qPCR of selected genes in U87-ST8Sia3 vs. U87-MG cell lines: OLIG2 (**a**), CHI3L1 (**b**), TIMP3 (**c**), TNFAIP2 (**d**), and TNFAIP6 (**e**). Results are shown as fold change from control with box plots. The lower and upper edges of the box represent the first and third quartile respectively, while a horizontal line within the box indicates the median and the red cross the mean. The vertical length of the box represents the Interquartil range (IQR). The most extreme sample values (within a distance of 1.5 IQR from the median) are the endpoints of the lines extending from the box, while the blue circles represent the outliers (1.5 IQR above 75th percentile) and far outliers (3 IQR above 75th percentile) respectively. *** *p* < 0.001, * *p* < 0.05.

Since we failed to identify a transcriptomic signature induced by neuraminidase in CSC lines, we performed a pan-transcriptomic analysis of U87-MG and U87-ST8Sia3 cultured in FCS-containing medium, in order to identify molecular mechanisms associated with A2B5 overexpression induced by ST8Sia3 in this cell model. The ratio list with a fold change strictly higher than 3 and a p-value strictly lower than 10^−5^ was established. Genes upregulated in U87-ST8Sia3 were annotated into functionally relevant categories (Table 1). Overall, significantly overexpressed 63 genes were identified in the U87-ST8Sia3 cell line. Most of them were involved in signal transduction, cell signaling, receptors and developmental processes (24% of the total genes), metabolism (20%) and cell structure and motility (16%). For the validation of RNA-seq results, using the RT-qPCR method, we chose to focus on five genes (*OLIG2*, *CHI3L1*, *TIMP3*, *TNFAIP2* and *TNFAIP6*) that were of high interest in GBMs as indicated in the literature and involved in proliferation, stemness and invasion since these functions are of the utmost importance in GBM biology and were reduced after neuraminidase administration. For each cell line, 10 independent samples were used for the quantification of mRNA levels of selected genes. As expected according to the RNA-seq results and as shown in Figure 4, *OLIG2* transcript was significantly overexpressed in U87-ST8Sia3 as compared to U87-MG (*p* < 0.001) (Figure 4a). RNA-seq results were also confirmed for *TIMP3* (*p* < 0.001) (Figure 4c), *TNFAIP2* (*p* < 0.001) (Figure 4d) and *TNFAIP6* (*p* < 0.05) (Figure 4e), and finally, *CHI3L1* was overexpressed in the U87-ST8Sia3 cell line, but the difference in expression between the two cell lines tested did not reach statistical significance (*p* = 0.121) (Figure 4b).

### 2.6. Neuraminidase Administration down Regulated OLIG2, CHI3L1, TIMP3, TNFAIP2 and TNFAIP6 Expression in CSC Lines

**Figure 5:**
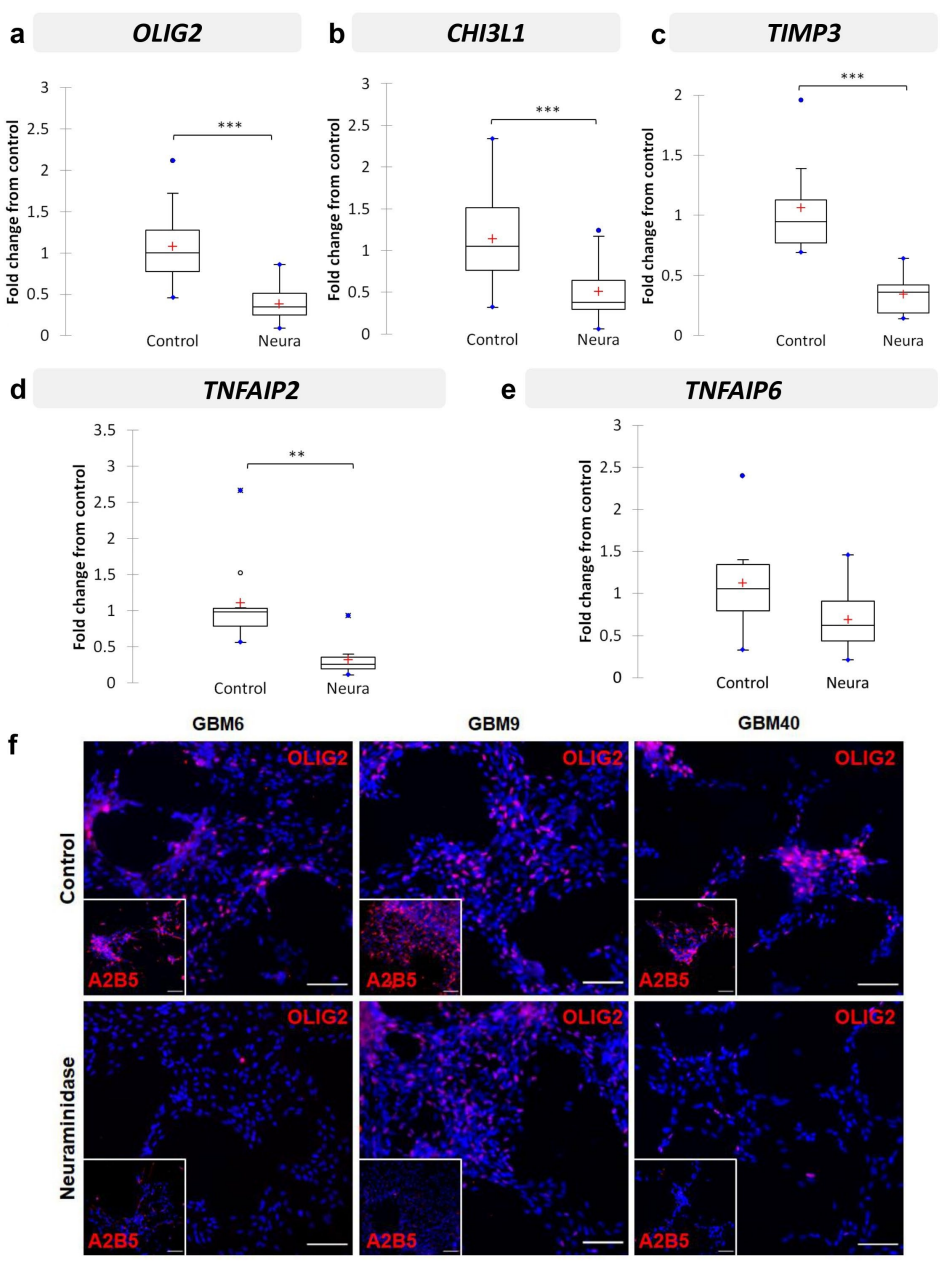
Validation by RT-qPCR of selected genes in CSC (GBM6, GBM9, GBM40, with or without neuraminidase (Neura) administration): *OLIG2* (**a**), *CHI3L1* (**b**), *TIMP3* (**c**), *TNFAIP2* (**d**), and *TNFAIP6* (**e**). Results are shown as fold change from control with box plots. The lower and upper edges of the box represent the first and third quartile respectively, while a horizontal line within the box indicates the median and the red cross the mean. The vertical length of the box represents the Interquartil range (IQR). The most extreme sample values (within a distance of 1.5 IQR from the median) are the endpoints of the lines extending from the box, while the black circles and the blue circles represent the outliers (1.5 IQR above 75th percentile) and far outliers (3 IQR above 75th percentile) respectively. Immunofluorescence detection of OLIG2 protein (**f**) in GBM6, GBM9 and GBM40 CSC lines in control cells (top panel) or after 24 h neuraminidase administration at EC50 (bottom panel). The small square shows the loss of A2B5 immunostaining after neuraminidase administration. Scale bar = 100 μm. *** *p* < 0.001, ** *p* < 0.01.

Since we aimed to search for changes in gene expression in A2B5-expressing cell lines after neuraminidase expression, we next analyzed the expression of the five selected genes *OLIG2*, *CHI3L1*, *TIMP3*, *TNFAIP2* and *TNFAIP6* in the three CSC lines treated or not with neuraminidase for 24 h, thus with enzymatically modified A2B5 expression levels. In addition, *NESTIN* and *PROM-1*, two precursor cell markers, were investigated. The results are reported in Figure 5. In all cases, neuraminidase administration induces significant down-regulation of *OLIG2* expression (*p* < 0.001) (Figure 5a), *CHI3L1* (*p* < 0.001) (Figure 5b), *TIMP3* (*p* < 0.001) (Figure 5c), *TNFAIP2* expression (*p* < 0.01) (Figure 5d), and with a tendency for *TNFAIP6* expression (*p* = 0.054) (Figure 5e). *NESTIN* and *PROM-1* precursor cell markers were also significantly down-regulated by neuraminidase (*p* = 0.001 and *p* = 0.007, respectively) (Supplementary Figure S2a,b). Details of the samples used and pooled for the analysis are reported in Supplementary Table S2. Moreover, we observed that *OLIG2* was the only gene for which the down-regulation induced by neuraminidase administration remained significant in each CSC line when observed individually (Figure S2c) (GBM6, *p* = 0.007; GBM9, *p* = 0.031; GBM40, *p* = 0.002). Therefore, in order to confirm the results obtained at the transcriptome level, at the proteome level, we chose to focus on OLIG2 protein expression only. By immunofluorescence, we confirmed the strong reduction in OLIG2 protein expression in each CSC line after neuraminidase administration in comparison to control (Figure 5f).

To go further, we took advantage of the organotypic slice model to analyze neuraminidase effects on tumor growth (U87-ST8Sia3 and GBM6 cell lines) within its cerebral microenvironment. The RNA extraction yield from biopsy punch tumor samples did not allow us to perform the analysis of the five genes of interest; we analyzed *OLIG2* and *CHI3L1*. In accordance with the previous analysis on in vitro U87-ST8Sia3 and GBM6 culture models, neuraminidase administration downregulated *OLIG2* and *CHI3L1* expression in the biopsy punch samples, but the results did not reach statistical significance (n = 3) (Supplementary Figure S3).

### 2.7. Neuraminidase Administration Induced Major Changes in Cell Surface Ganglioside Expression

Gangliosides were analyzed by hydrophilic interaction liquid chromatography coupled to electrospray ionization mass spectrometry (LC-ESI/MS) [21]. First, precursor ion scanning of the characteristic fragment of gangliosides, N-Acetylneuraminic acid, at *m*/*z* 290, was performed in the negative mode. Gangliosides corresponding to commercial standards were detected in cell lines, confirming their identification: GM3, GM2, GM1, GD2, GD3, GD1a, GD1b, GT1b and GQ1b. Only GM1 co-eluted with GD3, which could, however, be distinguished thanks to MS. A few additional minor peaks could also be detected in addition to major ganglioside classes. They did not correspond to commercial standards and required high-resolution mass spectrometry to be characterized. Unfortunately, it was not possible to perform this additional technique because of an insufficient amount of material.

**Figure 6:**
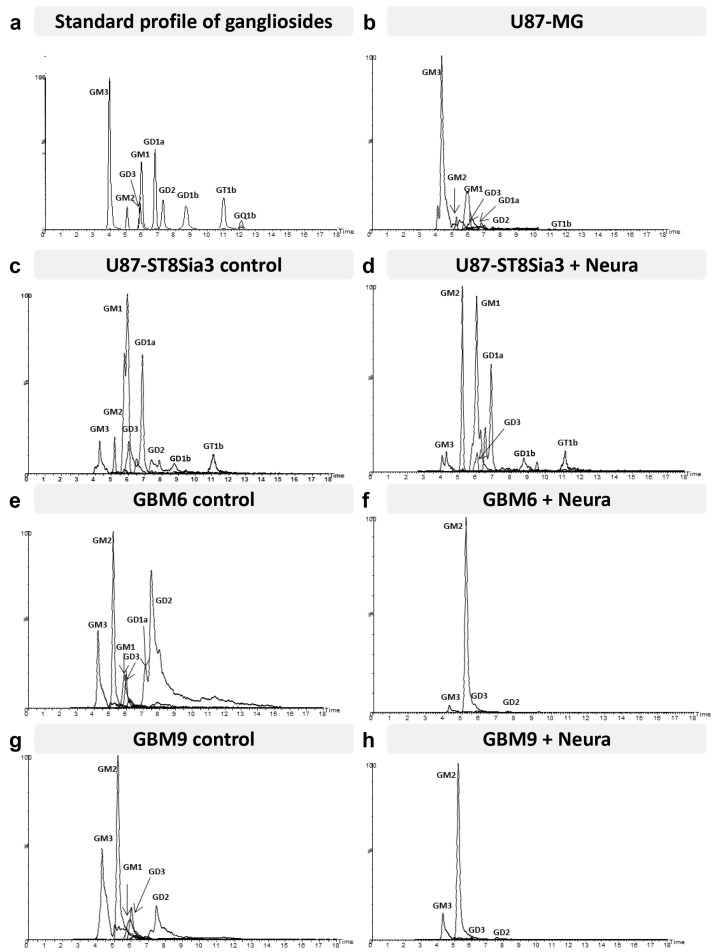
Hydrophilic interaction liquid chromatography–triple quadrupole mass spectrometry total ion chromatograms (TIC) of the standard mixture (GM3, GM2, GM1, GD3, GD2, GD1a, GD1b, GT1b and GQ1b) (**a**), U87-MG (**b**), U87-ST8Sia3 with or without neuraminidase (Neura) administration (**c**,**d**), GBM6 CSC line with or without neuraminidase administration (**e**,**f**) and GBM9 CSC line with or without neuraminidase administration (**g**,**h**). The mass spectrometer was operated in the negative precursor ion mode of the ion m/z 290 corresponding to N-acetyl neuraminic acid.

The proportions of the different molecular species detected, corresponding to different ceramide structures, were calculated for each ganglioside class independently, using the data obtained with the QqQ mass spectrometer operated in the negative SRM mode [21,22]. Figure 6 shows representative total ion chromatograms of a standard mixture of 9 ganglioside classes (GM3, GM2, GM1, GD3, GD2, GD1a, GD1b, GT1b and GQ1) (Figure 6a) and of different cell lines of interest treated or not by neuraminidase administration (U87-MG, U87-ST8Sia3, GBM6 and GBM9) (Figure 6b–h). The intensity of the different classes with each other cannot be compared. It depends on the ability of specific ganglioside to be fragmented, to form multi-charged ions [M-xH]x- and to be ionized in the apparatus. However, it is possible to compare ganglioside profiles between samples.

Regarding the ganglioside pattern, cell line specificities clearly appeared. The U87-MG cell line (Figure 6b) presented a wide diversity of gangliosides since seven different classes could be clearly detected and quantified: GM3, GM2, GM1, GD3, GD2, GD1a, and GT1b. GM3 and GM1 were present in the largest proportions in this cell line. In the U87-ST8Sia3 cell line (Figure 6c), GM3 is clearly reduced compared to U87-MG. We assume that it is used for the generation of other gangliosides of the a-series but also of GD3 (b-series), which is itself massively transformed into GT3 by the overexpressed ST8sia3 enzyme. Accordingly, we noted an increase in the rate of GD1a and GD3 in the U87-ST8Sia3 cell line compared to U87-MG.

The ganglioside pattern of the CSC lines was quite different from the U87 cell lines. In the GBM6 line (Figure 6e), we observed lower amounts of GM3 and GM1 but higher levels of di-sialo gangliosides GD2, GD3 and GD1a. The GBM9 cell line (Figure 6g) presented a profile quite close to the GBM6 cell line, especially for GM3, GM1, and GD3, but with a lower amount of GD2 and GD1a. Finally, neuraminidase administration induced in both CSC lines a drastic decrease in all classes of gangliosides except GM2 (Figure 6f,h). A decrease in gangliosides was also observed in the U87-ST8Sia3 cell line (Figure 6d).

## 3. Discussion

Our previous results supported the view that the A2B5 epitope plays a crucial functional role in the promotion of proliferation, migration, clonogenicity and tumorigenesis of GBM CSC. Moreover, the lentiviral delivery of shST8Sia3 in two CSC lines (GBM6 and GBM9) expressing a high level of A2B5 stopped their growth, highlighting that the A2B5 epitope sustained GBM CSC viability [18]. Therefore, neuraminidase that strongly decreases A2B5 expression at the CSC surface might be an attractive therapeutic target for human GBM but requires a better understanding of its action at the cellular level before using it to target human GBM. With this aim, we first confirmed that neuraminidase administration in the GBM organotypic slice model strongly decreased A2B5 expression and the size of the tumors generated after spheroid implantation. However, it is clear that it does not lead to regression of the tumor but rather to stabilization. This might be explained by the persistence of some A2B5-expressing cells after neuraminidase administration and/or the reappearance of A2B5 expression at the cell surface of some tumor cells. It is well known, however, that numerous drugs, especially temozolomide, display a transient efficacy in GBM, leading to tumor resistance and treatment failure (reviewed in [23]), and that this resistance might be secondary to the presence of GBM CSC [24]. Of interest, a previous report showed that adjuvant immunotherapy targeting O-acetyl GD2 ganglioside impaired temozolomide resistance driven by glioma-stem-like cells [25].

Then, we searched for a genetic signature induced by neuraminidase administration in the three A2B5 high CSC lines previously produced in our laboratory. We failed to identify such a genetic signature, and we learnt from the RNAseq performed that each cell line had its own transcriptomic signature and that this signature did not really change after neuraminidase administration, likely because the broad differences between the transcriptome and epigenetic signature of each CSC cell line (GBM6, GBM9 and GBM40) hide those induced by 24 h neuraminidase administration.

We also observed that neuraminidase administration did not induce a specific epigenetic signature. Again, DNA methylation profiling showed that each cell line displayed a similar DNA methylation profile to its neuraminidase counterpart. DNA methylation study also showed that the methylation profile of the U87-MG cell line and its derivatives were strikingly different from that observed in CSC lines. Moreover, the DKFZ human brain tumor classifier [26] was unable to provide a DNA methylation class (MC) for the U87-MG cell line, in contrast to the CSC lines that had an MC of GBM, *IDH*-wildtype. This result is in keeping with recent studies that have challenged the origin of the U87-MG cell line [27]. It is likely, however, that this cell line, which was set up many years ago [28], might have been derived.

Nevertheless, the U87-MG cell line, which is widely used, remains an attractive model, easy to manipulate for screening the role of some selected genes. Therefore, we used it to generate the U87-ST8Sia3 cell line in which the ST8Sia3 gene was stably overexpressed by lentiviral infection. We observed that the percentage of cells expressing A2B5 was high in U87-ST8Sia3 (80.2% ± 3.6), in comparison to the U87-MG (14.6% ± 6.9) or to the U87-ST8Sia3 cell line treated with neuraminidase (17.3 ± 7.8). By comparing the transcriptomic signature of the U87-ST8Sia3 cell line to that of U87-MG, we identified 63 genes significantly overexpressed in the U87-ST8Sia3 cell line. Among them, we selected five genes previously reported to be relevant in GBM: *OLIG2*, *CHI3L1*, *TIMP3*, *TNFAIP2* and *TNFAIP6*. We first confirmed by RT-qPCR that all these genes except one (*CHI3L1*) were overexpressed in the U87-ST8Sia3 cell line in comparison to U87-MG (*p* < 0.001 for *OLIG2*, *TIMP3* and *TNFAIP2* and *p* < 0.05 for *TNFAIP6*). Of the utmost interest, we further confirmed in the GBM CSC that neuraminidase administration drastically reduced the expression of the five selected genes (*p* < 0.001 for *OLIG2*, *CHI3L1* and *TIMP3*; *p* < 0.01 for *TNFAIP2* and *p* = 0.054 for *TNFAIP6*). Moreover, we further showed a strong reduction in OLIG2 protein expression by immunofluorescence in the three CSC lines after neuraminidase administration.

All these genes play a crucial role in stemness (*OLIG2*), invasion (*CHI3L1*, *TIMP3*, *TNFAIP2*) and proliferation (*TNFAIP2* and *TNFAIP6*), three major functions that were found to be sustained by A2B5 epitope expression [18].

*OLIG2* transcription factor is implicated in neurogenesis and gliogenesis [29] and is described as a key gene in gliomagenesis. Although the requirement of *OLIG2* in maintaining the growth of GBM cells is unknown, Ligon et al. suggested that it was essential in establishing xenografts [30], and Rich et al. reported that *OLIG2* expression was restricted to CSCs and was probably a proliferation regulator [31]. In another model, Bao et al. showed that targeting *L1CAM* expression in CSCs impaired cell growth and sphere formation and was associated with a decrease in *OLIG2* expression and apoptosis induction [6]. More recently, it was reported that *OLIG2* modulates growth factor signaling in two distinct populations of glioma stem-like cells depending on their *EGFR* or *PDGFRα* expression [32]. Of interest, in our study, the *OLIG2* gene was the only one that remained significant when each GBM CSC line was studied separately. Since these three cell lines exhibit a distinct transcriptomic profile, as previously reported for GBM6 and GBM9 [19], and epigenetic profile (present study), this result emphasizes that the strong decrease in *OLIG2* gene expression both at the transcriptome level but also at the protein level after neuraminidase administration might affect all GBM subtypes.

*TNFAIP2* is a primary response gene of TNFα, and high expression is associated with increased cell proliferation, migration, invasion and metastasis in various cancers including breast and esophagus cancers and gliomas [33,34,35]. Interestingly, it has been shown that in myeloma cells, *TNFAIP2* was significantly induced by cell adhesion to fibronectin [36], an extracellular matrix protein that is strongly overexpressed in GBM [37,38].

*TNFAIP6* was recently reported as a Hub gene associated with the progression of GBM by weighted gene co-expression network analysis [39]. Of interest also, *TNFAIP6* was identified among *ARL4C* and *MSN* as a neural progenitor cell-associated chemoradiotherapy resistance gene set for the prognosis of glioma [40]. Moreover, *TNFAIP6* also promotes invasion in various other cancers [41,42].

*CHI3L1* is overexpressed in many human cancers including GBM (reviewed in [43]). *CHI3L1* stimulates cell growth and proliferation in fibroblasts through the phosphorylation of MAPK and AKT signaling [44]. In GBM, *CHI3L1* regulates tumorigenesis by interrupting the pathways leading to apoptosis and by remodeling the extra-cellular matrix to create a good substrate for tumor growth [45]. It has also been recently reported that it promotes glioma progression via the NFKB signaling pathway and tumor microenvironment reprogramming [46].

*TIMP3* belongs to the tissue inhibitors of metalloproteinases (MMPs) that regulate the pericellular proteolysis of a vast range of matrix and cell surface proteins, generating simultaneous effects on the tumor architecture and cell signaling. TIMPs (1 to 4) are present in the extracellular matrix (ECM) in a soluble form, except for TIMP3, which is bound to ECM. All TIMPs inhibit MMP through reversible blockage, forming 1:1 stoichiometric complexes [47]. Among the members of the family, *TIMP3* silencing is usually associated with cancer progression or poor patient prognosis [47], but some studies also reported poor prognosis associated with high *TIMP3* expression [48]. Here, we found that *TIMP3* expression was reduced after neuraminidase administration and therefore might impair its function. Importantly, TIMP3 binds to MMP2 and MMP9 (reviewed in [49]), and we have previously reported that GBM MMP2 is expressed by tumor cells whereas MMP9 is expressed by tumor-infiltrative neutrophils. Moreover, in the AVAglio study [50], showed that the baseline plasma MMP9 level was predictive of bevacizumab efficacy in newly diagnosed GBM [51]. Whether a high TIMP3 level changes its binding to the ECM and/or induces disequilibrium between the MMP2 and MMP9 level in GBM, leading to adverse effects, remains unknown. However, it is obvious that the regulation of the TIMPS family in cancer remains complex, leading to functional disparities depending on the model used [52].

Furthermore, we learnt from this study that the pattern of ganglioside expression revealed by liquid chromatography–mass spectroscopy was strikingly different in the U87-MG cell line versus the CSC line. The U87-MG cell line was characterized by a high expression of GM3 ganglioside, as previously reported [53]. Importantly, ST8Sia3 overexpression in the U87-MG cell line induced a switch from the GM3 ganglioside towards other gangliosides of the a-series (GM2, GM1) and to b-series gangliosides (GD3, GD2 and GD1b). Unfortunately, this technique was not fully appropriate for the detection of c-series gangliosides (which are the main A2B5 carriers), but because of the switch towards b-series induced by ST8Sia3, it is likely that some c-series gangliosides might be included in the ‘non-identified’ ganglioside compound observed in the U87-ST8Sia3 cell line. In contrast to what was observed in the U87-MG cell line, GBM6 and GBM9 CSC lines displayed a similar ganglioside profile characterized by the overexpression of GM2 and b-series gangliosides, a pattern reminiscent of that described in the U87-ST8Sia3 cell line. Of importance, we observed that neuraminidase administration in both CSC lines drastically reduced ganglioside expression at their cell surface, except for GM2. This might be explained by the action of neuraminidase, which preferentially hydrolyses α2-3 and α2-8 terminal sialyl linkages but is ineffective on the α2-3 inner sialic acid branch (characterizing GM1 and GM2) in accordance with the fact that GM2 was not affected by the enzyme [54,55]. These overall changes after neuraminidase administration were less obvious in the U87-ST8Sia3 cell line, likely because of the constitutive synthesis of b-series gangliosides induced by ST8Sia3. Several studies have reported that GD2 and GD3 are highly expressed in GBM as well as several glycosyl transferases involved in their biosynthesis [56,57,58]. Moreover, GD3 synthase appeared as a key driver for GBM cancer stem cell maintenance and tumorigenesis [57]. Therefore, neuraminidase, which strongly affects sialic acid expression carried by complex gangliosides, induces major changes in cell surface glycosylation of CSC lines and consequently affects cell–cell and cell matrix interaction, with a major impact on downstream processes [59]. Moreover, the sialylation status is exploited by tumors to evade both innate and adaptative immune destruction. Cell surface sialylation is recognized by complement factor H, and sialic acid-binding immunoglobulin-like lectin (Siglec) acts as a major checkpoint for immune response in the central nervous system (reviewed in [60]). Moreover, it has also been shown that sialic acid blockade suppresses tumor growth by enhancing T-cell-mediated tumor immunity [61]. Therefore, neuraminidase that strongly affects cell surface ganglioside by inducing less sialylated ones might be also considered as a strategy for GBM immunotherapy.

## 4. Materials and Methods

### 4.1. Cell Lines, Culture Conditions and Reagents Used

U87-MG (American Type Culture Collection) and U87-ST8Sia3 (described in [18]) cell lines were cultured as an adherent monolayer (or in suspension for spheroid formation) in Dulbecco’s modified Eagle’s medium with 10% heat-inactivated fetal calf serum (FCS, ThermoFisher Scientific, Courtaboeuf, France), 50 U/mL penicillin and 50 µg/mL streptomycin (ThermoFisher Scientific, Courtaboeuf, France), at 37 °C in 5% CO_2_ humidified atmosphere. GBM6, GBM9 and GBM40 A2B5-positive CSC lines, established at our laboratory from human GBM tumor samples, were cultured as floating spheres in a stem-cell permissive serum-free medium as previously described [18]. Stable U87-ST8Sia3-GFP, GBM6-GFP and GBM9-GFP cell lines were obtained by transfection with GFP-C1 (Takara Bio Europe SAS, Saint-Germain-en-Laye, France) and geneticin selection. For spheroid formation, 5000 cells/well from U87-ST8Sia3-GFP, GBM6-GFP and GBM9-GFP cell lines were seeded in an appropriate medium with 20% methylcellulose on U-bottom 96-well plates for 24 h before their use for organotypic slice experiment. All cell lines were maintained at 37 °C in a 5% CO_2_-humidified atmosphere.

For in vitro treatment experiments, all cells were cultured at 37 °C with neuraminidase from Clostridium Perfringens (Merck, Saint Quentin Fallavier, France) at their respective EC50 (U87-MG 0.91 U/mL; U87-ST8Sia3 and U87-ST8Sia3-GFP: 1 U/mL; GBM6 and GBM6-GFP: 0.98 U/mL; GBM9 and GBM9-GFP: 0.56 U/mL and GBM40: 0.96 U/mL [18]. Clostridium perfringens was used by Eisenbarth et al. [10] when they described the A2B5 antibody. They demonstrated that this neuraminidase destroyed the sialic acids that are required for the A2B5 antibody’s binding.

### 4.2. Stainings

Flow cytometry was performed using MACSQuant^®^ 10 (Miltenyi Biotec SAS, Paris, France) on dissociated cells using A2B5 (mouse APC-IgM, clone 105, Miltenyi Biotec SAS) following the manufacturer’s instructions. Data were analyzed using FlowJo software (10.8.1, Becton Dickinson & Company, Tree Star, Inc., Ashland, OR, USA).

For immunofluorescence microscopy, U87-ST8sia3 cells were grown as a monolayer on glass coverslips in 24-well plates in DMEM with 10% FCS. The primary antibodies A2B5 (mouse IgM, clone 105, 1/1000, kindly provided by G. Rougon, Marseille, France) and OLIG2 (goat IgG, 1/50, Bio-Techne SAS, Noyal Châtillon sur Seiche, France) were incubated for 1 h at room temperature followed by fluorochrome-conjugated secondary antibodies Alexa fluor 568 anti-mouse IgM and Alexa fluor 568 anti-goat IgG (Molecular Probes, Eugene, OR, USA) incubated at 2 μg/mL for 1 h at room temperature together with Hoechst 33342 (1/1000, Merck, Saint Quentin Fallavier, France). All the images were obtained using a Zeiss AXIO-Observer Z1 microscope (Carl Zeiss SAS, Marly-le-Roi, France).

### 4.3. GBM Organotypic Slice Culture Model

Six-week-old athymic female nude mice (Envigo, Gannat, France) were anesthetized and transcardially perfused with PBS. After the mice were euthanized, brains were dissected, placed in cold PBS containing 100 U/mL penicillin, 100 mg/mL streptomycin and 0.25 µg/mL amphotericin B (Merck, Saint Quentin Fallavier, France), then glued between agarose blocks onto the vibratome disc (VT 1200 S, Leica, Nanterre, France) with Roti Coll1 glue (0258.1 Carl Roth, Lauterbourg, France). The vibratome chamber filled with cold PBS containing 100 U/mL penicillin and 100 mg/mL streptomycin and 250 μm thick sections were prepared and transferred to petri dishes filled with cold PBS containing 100 U/mL penicillin, 100 mg/mL streptomycin and 0.25 µg/mL amphotericin B. Millipore inserts (PICM 03050, Merck, Saint Quentin Fallavier, France were placed in six-well plates filled with a 1.2  mL slice culture medium consisting of 50% MEM, 25% Hanks’ balanced salt solution (HBSS), 25% normal horse serum (NHS), 0.2 mM glutamine, 100 U/mL penicillin, 100 mg/mL streptomycin (Sigma, Munich, Germany), 0.25 µg/mL amphotericin B and 4.5 mg/mL glucose, using a Rotilabo-embryo spoon (TL85.1, Carl Roth, Lauterbourg, France). A maximum of three slices were placed per insert. One day after, a spheroid with a diameter of approximately 250 µm from each GBM cell line was collected using a micropipette in a volume of 50 µL per well and implanted by placing it on top of a slice. The co-culture was maintained at 37 °C, 5% CO_2_, 95% humidity. At days 1, 3, 6, 8, 10 and 15 after spheroid implantation, organotypic slices were treated with neuraminidase at EC50 corresponding to the different implanted tumor cell types or PBS (control). When necessary, tumors were taken out from the slices on day 16 using a 2 mm biopsy punch (Kai Medical, Seki, Japan), dissociated with accutase (Merck, Saint Quentin Fallavier, France) and analyzed by flow cytometry for A2B5 expression. In addition, some tumors were taken out, washed with PBS and quickly frozen at −80 °C before total RNA extraction.

Neuraminidase’s effect on tumor regrowth after surgical removal of the tumors generated by U87-ST8Sia3-GFP or GBM6-GFP spheroids at 16 days post-implantation was also analyzed. For this purpose, organotypic slices were treated with neuraminidase at EC50 or vehicle (PBS) at days 1, 4 and 6 post tumor resection (day 0). At day 7, the newly formed tumors were removed, dissociated with accutase (Merck, Saint Quentin Fallavier, France) and analyzed by flow cytometry for A2B5 expression.

Images were acquired using a Zeiss AXIO-Observer Z1 microscope (Carl Zeiss SAS, Rueil-Malmaison, France) with a 4× fixed objective in the GFP+ channel. Images were automatically stitched together using the Tile Scan mode of the microscope. The total size area of each tumor generated by the spheroids was measured by Image J (NIH, Bethesda, MD, USA) by connecting the farthest invaded cells.

### 4.4. DNA Methylation Analysis

#### 4.4.1. DNA Extraction and Quantification

10^6^ U87-MG, U87-ST8Sia3 or 24 h neuraminidase-treated U87-ST8Sia3 cells as well as 7-day-old GBM6 and GBM9 CSC spheres treated or not with neuraminidase for 24 h were collected, washed with PBS, and centrifuged in 1.5 mL tubes. Pellets were finally stored at −80 °C until use.

DNA was extracted from each cell line, quality checked, quantified as well as converted by bisulfite as previously described [62]. The DNA was then processed using the Illumina Infinium HumanMethylation EPIC Bead-Chip array (Illumina, San Diego, CA, USA) according to the manufacturer’s instructions. iScan control software v3.4 (Illumina, San Diego, CA, USA) was used to generate raw data files from the BeadChip in .idat format, analyzed using GenomeStudio version 2.0 (Illumina, San Diego, CA, USA), and checked for quality measures according to the manufacturer’s instructions.

#### 4.4.2. DNA Methylation Data Processing

The .idat files were uploaded to the online and publicly available CNS tumor DNA methylation classifier (11b4 and 12.5 versions) from the German Cancer Research Center (Deutsches Krebsforschungszentrum, DKFZ) at https://www.molecularneuropathology.org (version 6.9.2, Heidelberg, Germany) and a report for every tumor was generated, providing prediction scores for methylation classes (MC) and chromosomal copy-number-variation (CNV) plots. The scores were integrated into the histopathological findings according to the recommendations from Capper et al. [26,63]. A prediction score >0.9, in v12.5 or >0.84 in v11b4, was considered high and relevant for diagnosis.

Additional analyses were performed in R studio (v4.0.2). Raw signal intensities were obtained from .idat files using the minfi Bioconductor package (v1.34.0). Background correction and dye-bias correction were performed on each sample. Filtering criteria of probes were the removal of probes targeting X or Y chromosomes and the removal of probes containing single-nucleotide polymorphisms. Hierarchical clustering was performed using the Complex Heatmap package (2.4.3). Clustering of beta values from methylation arrays was performed based on the Euclidean distance with a ward algorithm. Methylation heatmaps show only the most variable probes (SD > 0.20).

Chromosomal CNV was searched for by visual inspection of CNV profiles generated by the molecularneuropathology.org platform as described [63,64].

### 4.5. Transcriptomic Analysis by RNA-Sequencing and Differential Gene Expression Analysis

#### 4.5.1. Transcriptomic Analyses by RNA-Sequencing

Two transcriptomic analyses were performed using two Illumina sequencing platforms. The first one, ProfileXpert (Lyon, France), was instrumental for RNA-seq from GBM6, GBM9 and GBM40 CSC lines (treated or not with neuraminidase), whereas in the second one, GBiM platform (Marseille, France), RNA-seq from U87-MG, and U87-ST8Sia3 was performed. Briefly, the total RNA was isolated and qualified, and polyA+ mRNA purification was performed using Qiamp (Qiagen SAS, Courtaboeuf, France), Agilent Bioanalyzer 2100 (Agilent Technologies, Les Ulis, France) and polyT oligo-attached magnetic beads. After two rounds of enrichment for PolyA+ mRNA and thermal fragmentation, cDNA was synthesized using superscript II and random primers, followed by second-strand cDNA synthesis, end repair process, adenylation of 3’ ends and ligation of the adapters. The products were then purified and enriched with 15 cycles of PCR to create the cDNA library. Libraries were quantified by qPCR using the KAPA Library Quantification Kit for Illumina Libraries (Roche Applied Science, Meylan, France), and profiles were assessed using the DNA High Sensitivity LabChip Kit (Agilent Technologies, Les Ulis, France) on an Agilent Bioanalyzer 2100 (Agilent Technologies, Les Ulis, France). Libraries were sequenced on an Illumina NextSeq 500 using a cartridge of the NextSeq 500/550 High Output v2 kit (150 cycles) (Illumina, San Diego, CA, USA).

#### 4.5.2. Differential Gene Expression Analyses

The raw sequencing data were first converted into FastQ files, which contain 75 nt single-end reads. This conversion was performed using Illumina bcl2fastq software version 2.17.1.14 by allowing for one mismatch in the barcodes when demultiplexing the reads. The quality of the reads was then assessed using FastQC version 0.11 [65]. In order to ensure high-quality data, any low-quality bases and adapters were removed from the reads using Cutadapt version 1.9.1 [66]. The quality threshold score used for this process was set to 30, and the minimum length of the reads was set to 50 nt after trimming. Next, the reads were pseudo-aligned to the GRCh38 transcriptome using kallisto version 0.44.0 [67]. This step enabled the quantification of the transcript abundances within the samples. Counts were then normalized and analyzed for differential gene expression using DESeq2 version 1.26.0 [68]. Differentially expressed genes were identified as those with a fold change of log2 (fc) ≥ log2 (1.5) and a *p*-value < 0.05, with *p*-values being adjusted using the Benjamini and Hochberg method. Genes that were identified as differentially expressed were classified as being up-regulated or down-regulated based on the fold-change values. In order to obtain a short list of relevant genes from the data generated by the GBiM platform (Marseille, France), we differentially selected genes that showed a fold change strictly higher than 3 and *p* value strictly lower than 10^−5^; http://www.genecards.org and http://www.genome.jp/kegg/pathway.html were used for gene analysis.

### 4.6. Real-Time Quantitative PCR Analysis (RT-qPCR)

A measure of 5 × 10^5^ U87-MG, U87-ST8Sia3, as well as a flask of 7-day-old GBM6 and GBM9 spheres (corresponding to 3–5 × 10^5^ cells) treated or not with neuraminidase for 24 h were collected and washed with PBS. Total RNA extraction and RT-qPCR from either frozen cell pellets or biopsy punch tumors were performed as previously described [69]. Quantification and purity determination were performed using a DS-11 spectrophotometer (Denovix, Wilmington, NC, USA). PCR conditions for 18S (reference gene), *OLIG2*, *CHI3L1*, *TNFAIP2*, *TNFAIP6*, *TIMP3*, *PROM-1* and *NESTIN* were 5 min at 95 °C, followed by 45 cycles of 15 s at 95 °C and 30 s at the respective annealing temperature, and are summarized in Supplementary Table S3 as well as primer sequences.

Gene expression was determined by using real-time quantitative polymerase chain reaction (RT-qPCR) analysis. The target genes’ Ct values were normalized with 18S Ct. The calculation was based on the 2−ΔΔCT method [70]. The fold change values of all samples were calculated as compared to their respective control samples. The number of samples analyzed for each cell line as well as the number of samples analyzed after punch removal is reported in Supplementary Table S2.

### 4.7. Ganglioside Expression in GBM Cell Lines

For ganglioside expression analysis, 2.10^6^ U87-MG or U87-ST8SIA3 cells or 7 days GBM6 and GBM9 spheres (corresponding to 1–2.10^6^ cells) were treated with neuraminidase or PBS for 24 h. Cells were then collected, washed with PBS, and centrifuged, and pellets were finally stored at −80 °C until use. Chloroform (CHCl_3_) was obtained from SDS (France). Ammonium acetate, acetonitrile (CH_3_CN), methanol (CH_3_OH) and water (H_2_O) of Optima LC/MS grade were all from Fisher Scientific (Illkirch, France). Commercially available ganglioside standards from natural sources (bovine or human) were obtained from Matreya LLC (State College, PA, USA).

The total lipids were extracted and purified according to Khoury et al. [71]. Liquid chromatography was performed using an Aquity UPLC H-Class PLUS system (Waters, Saint-Quentin-en-Yvelines, France). Separation of gangliosides was achieved under hydrophilic interaction liquid chromatography conditions according to Sibille et al. [21]. The triple quadrupole (QqQ) mass spectrometer (Xevo TQ-S micro, Waters, Saint-Quentin-en-Yvelines, France) equipped with a heated electrospray ionization source was operated in negative ion mode. For characterization, collision-induced dissociation of each deprotonated molecule was performed in the negative mode. An abundant product ion at *m*/*z* 290 corresponding to a characteristic N-Acetylneuraminic acid (sialic acid) fragment was obtained from [M-xH]x- ions of the different ganglioside molecular species. This fragment was used for precursor ion scanning, whereby the [M-xH]x- ions of gangliosides were specifically detected (Signal/Noise > 3). For quantification, data were acquired using monitoring reaction monitoring (MRM). The precursor and product ion pairs for the MRM analysis were selected based on the precursor ion scanning, but some species were not considered in SRM because they stood below the quantification limit (Signal/Noise < 10). The proportion of each molecular species of a specific ganglioside class was calculated as the ratio of its peak area to the sum of all detected peak areas in this class, every ganglioside class being considered separately. For more information about the developed analytical methods, see Masson et al. [22].

### 4.8. Statistical Analysis

The non-parametric Mann–Whitney test was conducted using the GraphPad 5.0 statistical software to analyze flow cytometry, tumor areas and differential gene expression results. All statistical tests were two-sided, and the threshold for statistical significance was *p* < 0.05. Significances: *** *p* < 0.001, ** *p* < 0.01, * *p* < 0.05.

## 5. Conclusions

In this paper, we have reported that neuraminidase administration in an organotypic slice model reduces tumor size as well as regrowth after removal but is not associated with a transcriptomic or epigenetic signature, although a strong decrease in gene expression involved in stemness, invasion and proliferation occurred. Moreover, neuraminidase administration also induces major changes in cell surface ganglioside expression and therefore sialylation that might influence the interactions between GBM tumor cells and the microenvironment. Although very promising, numerous experiments must be carried out before including neuraminidase in the GBM therapeutic arsenal, especially as local treatment, likely in combination with other drugs.

These include, among others, the measurement of the long-term action of neuraminidase, stabilization of the enzyme, and combination with other drugs such as temozolomide. In addition, given the role of sialic acids in both innate and adaptative immune responses, dedicated experiments must be carried out to analyze the role of neuraminidase in these processes.

## Figures and Tables

**Table 1:** Significantly overexpressed genes in U87-ST8Sia3 cells relative to U87-MG cells. Significantly overexpressed genes (>3-fold change, *p*-value > 10^−5^) were annotated into functionally relevant categories. http://www.genecards.org and http://www.genome.jp/kegg/pathway.html were used for gene analysis.

Category	Gene Symbol	Fold Change	*p Value*	Description
Signal transduction	*TNFAIP6*	6.23	7.88 × 10^−14^	TNF-Alpha-Induced Protein 6
Cell signalling	*PLEKHA1*	5.58	4.97 × 10^−12^	Pleckstrin Homology Domain Containing A1
Receptors	*WDFY1*	5.28	1.76 × 10^−11^	WD Repeat and FYVE Domain Containing 1
	*RNF139*	5.05	1.19 × 10^−10^	Ring Finger Protein 139
	*CSNK2A1*	4.99	1.21 × 10^−10^	Casein Kinase 2 Alpha 1
	*IL1B*	4.52	2.54 × 10^−09^	Interleukin 1 Alpha
	*TOLLIP*	4.10	3.63 × 10^−08^	Toll Interacting Protein
	*ADGRE1*	3.86	2.75 × 10^−07^	Adhesion G Protein-Coupled Receptor E1
	*TMEM14C*	3.55	1.10 × 10^−06^	Transmembrane Protein 14C
	*CIR1*	3.53	1.23 × 10^−06^	Corepressor Interacting With RBPJ, 1
	*RASA2*	3.52	1.56 × 10^−06^	RAS P21 Protein Activator 2
	*ROS1*	3.50	1.38 × 10^−06^	ROS Proto-Oncogene 1, Receptor Tyrosine Kinase
	*IGFBP4*	3.16	8.39 × 10^−06^	Insulin-Like Growth Factor Binding Protein 4
	*PTBP3*	3.09	1.34 × 10^−05^	Polypyrimidine Tract Binding Protein 3
	*TNFAIP2*	3.01	1.90 × 10^−05^	TNF-Alpha-Induced Protein 2
Transcription	*MAFF*	5.28	3.87 × 10^−11^	MAF BZIP Transcription Factor F
Translation	*MRTO4*	4.57	2.86 × 10^−09^	MRT4 Homolog, Ribosome Maturation Factor
	*MED21*	3.74	4.11 × 10^−07^	Mediator Complex Subunit 21
	*CHRAC1*	3.70	4.96 × 10^−07^	Chromatin Accessibility Complex 1
	*POLR2J*	3.57	1.67 × 10^−06^	RNA Polymerase II Subunit J
	*RPS17L*	3.48	1.42 × 10^−06^	Ribosomal Protein S17
	*ZNF721*	3.42	7.16 × 10^−06^	Zinc Finger Protein 721
	*KANSL3*	3.29	4.50 × 10^−06^	KAT8 Regulatory NSL Complex Subunit 3
	*RPS7*	3.22	6.15 × 10^−06^	Ribosomal Protein S7
	*TSC22D1*	3.10	1.14 × 10^−05^	TSC22 Domain Family Member 1
	*OLIG2*	3.05	3.27 × 10^−05^	Oligodendrocyte Transcription Factor 2
	*TBX3*	3.04	1.85 × 10^−05^	T-BOX 3
Cell cycle	*G0S2*	5.56	2.52 × 10^−12^	G0/G1 Switch 2
Mitosis	*PCNP*	4.42	4.42 × 10^−09^	PEST Proteolytic Signal Containing Nuclear Protein
	*INSIG1*	4.24	1.22 × 10^−08^	Insulin-Induced Gene 1
	*CSNK1G1*	3.35	3.31 × 10^−06^	Casein Kinase 1 Gamma 1
Cell structure	*MAP1LC3B*	7.46	8.77 × 10^−18^	Microtubule-Associated Protein 1 Light Chain 3 Beta
Motility	*IGFN1*	5.66	6.48 × 10^−12^	Immunoglobulin-Like Fibronectin Type III Domain Containing 1
	*PDLIM2*	5.54	1.45 × 10^−11^	PDZ And LIM Domain 2
	*FIBCD1*	5.36	2.05 × 10^−11^	Fibrinogen C Domain Containing 1
	*LASP1*	4.90	2.03 × 10^−10^	LIM And SH3 Protein 1
	*CHI3L1*	4.71	6.31 × 10^−10^	Chitinase 3 Like 1
	*TWF1*	3.62	6.12 × 10^−07^	Twinfilin Actin Binding Protein 1
	*P4HA1*	3.27	4.46 × 10^−06^	Prolyl 4-Hydroxylase Subunit Alpha 1
	*TIMP3*	3.26	1.11 × 10^−05^	TIMP Metallopeptidase Inhibitor 3
	*KRT8*	3.04	1.76 × 10^−05^	Keratin 8
Mitochondria	*ISCA2*	3.61	8.91 × 10^−07^	Iron-Sulfur Cluster Assembly 2
Metabolism	*PRDX1*	6.69	3.03 × 10^−15^	Peroxiredoxin 1
	*CHST1*	5.64	7.10 × 10^−12^	Carbohydrate Sulfotransferase 1
	*PDXK*	5.36	1.00 × 10^−11^	Pyridoxal Kinase
	*HSD11B1*	4.88	8.27 × 10^−10^	Hydroxysteroid 11-Beta Dehydrogenase 1
	*HS3ST2*	4.65	2.33 × 10^−09^	Heparan Sulfate-Glucosamine 3-Sulfotransferase 2
	*COLGALT1*	4.49	2.71 × 10^−09^	Collagen Beta(1-O)Galactosyltransferase 1
	*PSMB7*	4.04	1.03 × 10^−07^	Proteasome Subunit Beta 7
	*NHLRC2*	3.97	8.57 × 10^−08^	NHL Repeat Containing 2
	*SLC44A1*	3.92	1.01 × 10^−07^	Solute Carrier Family 44 Member 1
	*DNAJB2*	3.90	1.20 × 10^−07^	DnaJ Heat Shock Protein Family (Hsp40) Member B2
	*AOC2*	3.69	1.37 × 10^−06^	Amine Oxidase, Copper Containing 2
	*ACSS2*	3.49	1.34 × 10^−06^	Acyl-CoA Synthetase Short Chain Family Member 2
	*AKR1C1*	3.44	1.92 × 10^−06^	Aldo-Keto Reductase Family 1 Member C1
Transport	*SCOC*	7.50	1.83 × 10^−17^	Short Coiled-Coil Protein
	*SLC25A42*	4.23	2.81 × 10^−08^	Solute Carrier Family 25 Member 42
	*SLC39A1*	3.66	4.84 × 10^−07^	Solute Carrier Family 39 Member 1
	*RAB5C*	3.65	1.11 × 10^−06^	RAB5C, Member RAS Oncogene Family
Others	*CCDC117*	4.24	1.74 × 10^−08^	Coiled-Coil Domain Containing 117
	*KIAA1755*	3.72	8.03 × 10^−07^	Unknown
	*ICOSLG*	3.48	1.91 × 10^−06^	Inducible T-Cell Costimulator Ligand
	*BST2*	3.46	3.74 × 10^−06^	Bone Marrow Stromal Cell Antigen 2

## Data Availability

The data presented in this study are available on request from the corresponding author.

## Supplementary Materials

The following supporting information can be downloaded at: https://www.mdpi.com/article/10.3390/ijms241411645/s1.
