# Supplementary material for: Deciphering the Action of Neuraminidase in Glioblastoma Models
Source: Int J Mol Sci. 2023 Jul 19;24(14):11645. doi: 10.3390/ijms241411645 (PMC10380381; doi:10.3390/ijms241411645)
Supplement: Supplementary file 1 [file ijms-24-11645-s001.zip › ijms-2476155_Supplementary Figures.pptx]

## Slide 1
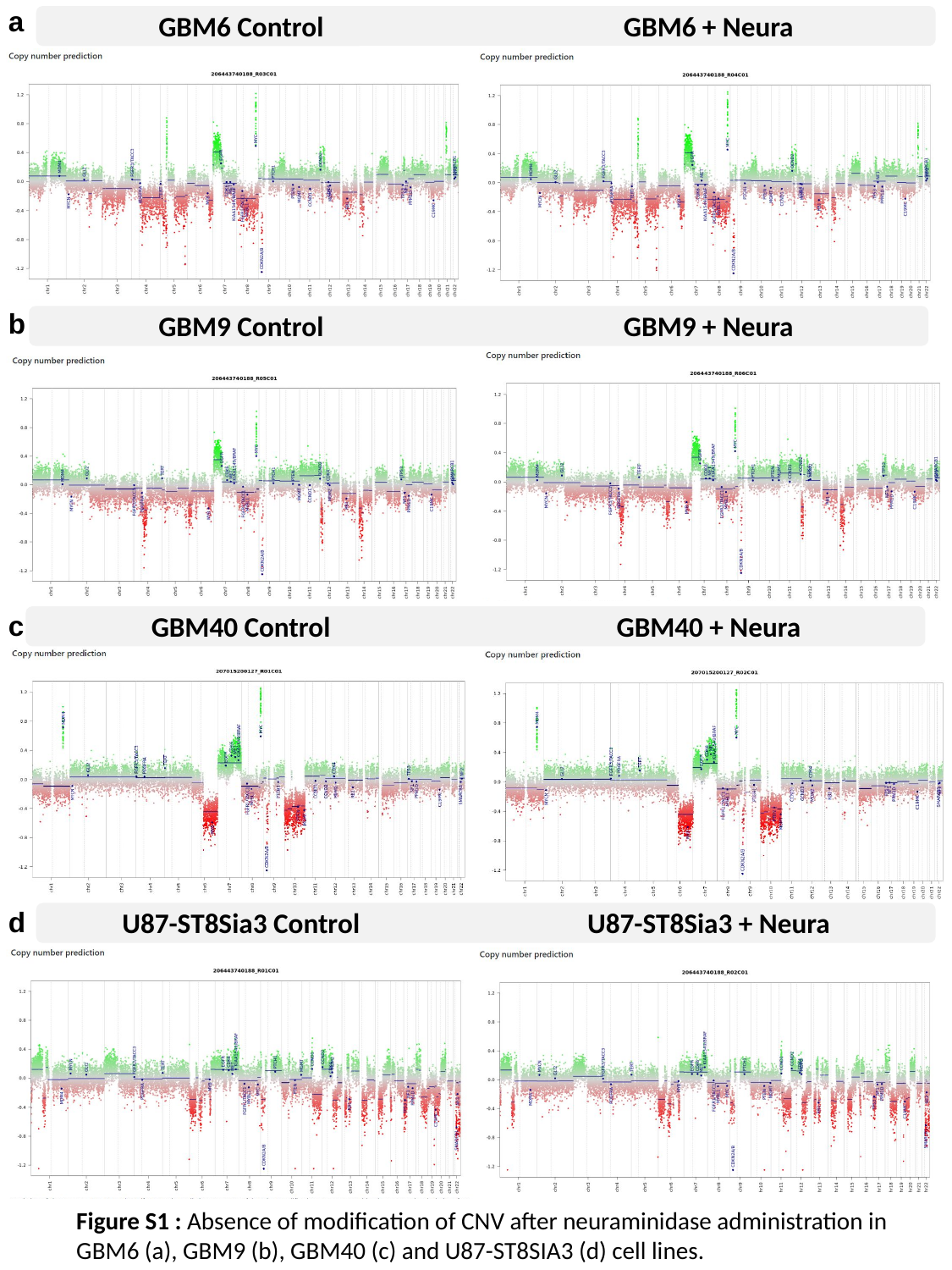

a
GBM6 Control
GBM6 + Neura
GBM9 Control
GBM9 + Neura
b
GBM40 Control
GBM40 + Neura
c
U87-ST8Sia3 Control
U87-ST8Sia3 + Neura
d
Figure S1 : Absence of modification of CNV after neuraminidase administration in GBM6 (a), GBM9 (b), GBM40 (c) and U87-ST8SIA3 (d) cell lines.

## Slide 2
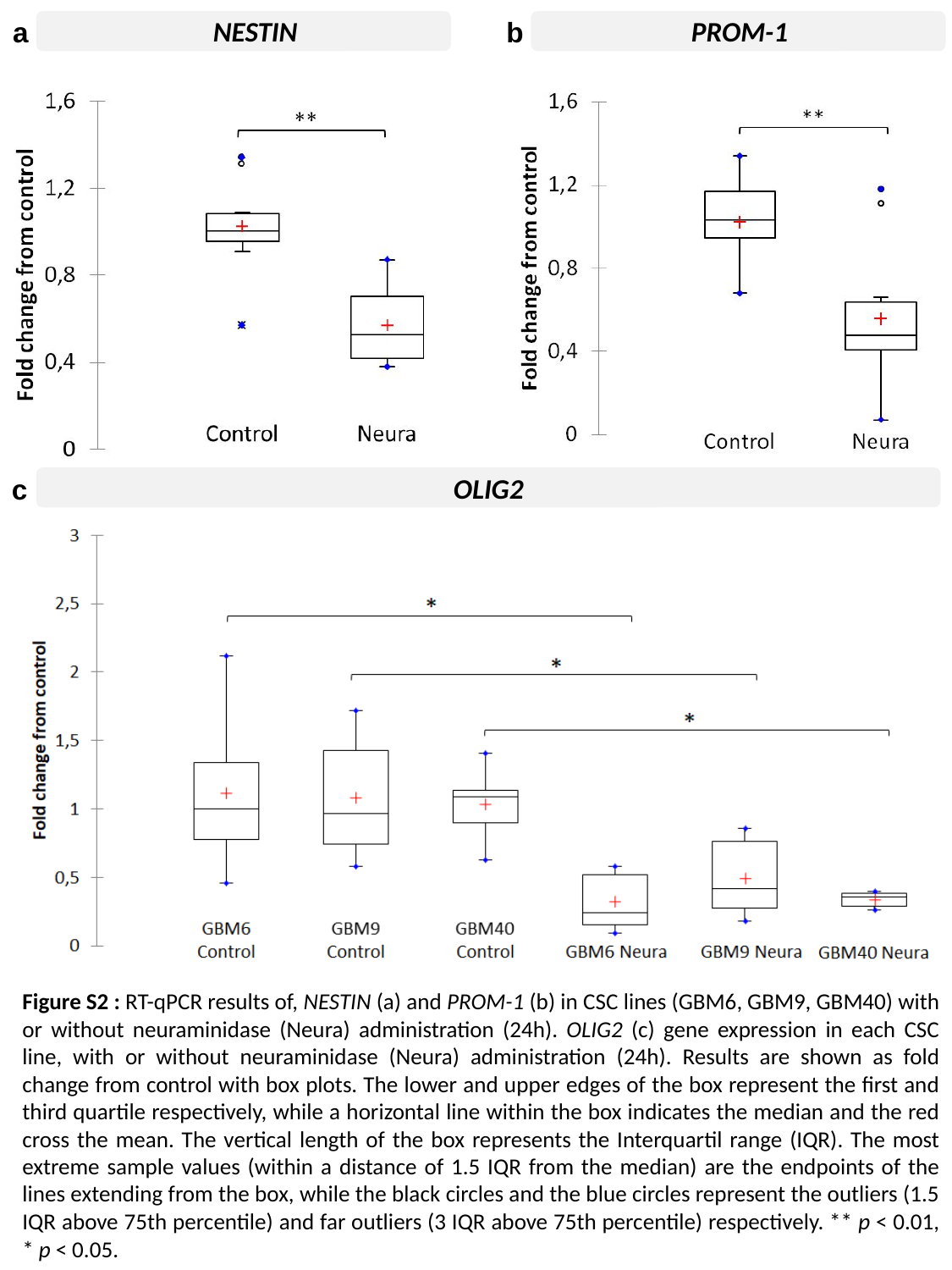

NESTIN
PROM-1
a
b
OLIG2
c
Figure S2 : RT-qPCR results of, NESTIN (a) and PROM-1 (b) in CSC lines (GBM6, GBM9, GBM40) with or without neuraminidase (Neura) administration (24h). OLIG2 (c) gene expression in each CSC line, with or without neuraminidase (Neura) administration (24h). Results are shown as fold change from control with box plots. The lower and upper edges of the box represent the first and third quartile respectively, while a horizontal line within the box indicates the median and the red cross the mean. The vertical length of the box represents the Interquartil range (IQR). The most extreme sample values (within a distance of 1.5 IQR from the median) are the endpoints of the lines extending from the box, while the black circles and the blue circles represent the outliers (1.5 IQR above 75th percentile) and far outliers (3 IQR above 75th percentile) respectively. ** p < 0.01, * p < 0.05.

## Slide 3
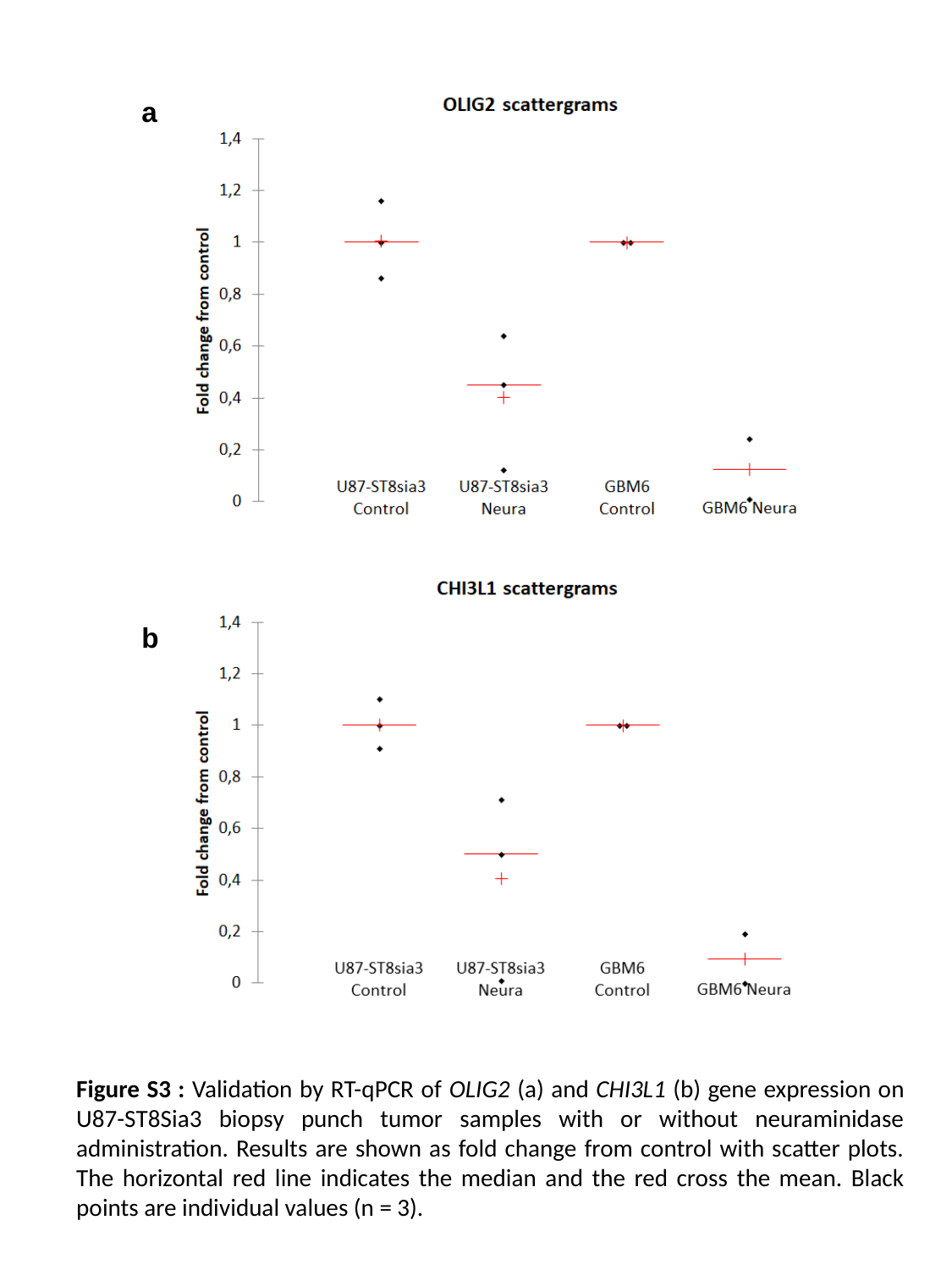

a
b
Figure S3 : Validation by RT-qPCR of OLIG2 (a) and CHI3L1 (b) gene expression on U87-ST8Sia3 biopsy punch tumor samples with or without neuraminidase administration. Results are shown as fold change from control with scatter plots. The horizontal red line indicates the median and the red cross the mean. Black points are individual values (n = 3).
